# Disability, discrimination and death: is it justified to ration life saving treatment for disabled newborn infants?

**DOI:** 10.1007/s40592-014-0002-y

**Published:** 2014-10-14

**Authors:** Dominic Wilkinson, Julian Savulescu

**Affiliations:** 1 Faculty of Philosophy, Oxford Uehiro Centre for Practical Ethics, University of Oxford, Suite 8, Littlegate House, St Ebbes St, Oxford, OX1 1PT UK; 2 Discipline of Obstetrics and Gynaecology, Robinson Institute, University of Adelaide, Adelaide, SA Australia; 3 John Radcliffe Hospital, Oxford, UK

**Keywords:** Neonatal intensive care, Resource allocation, Clinical ethics, Withdrawing treatment, Disability, Quality of life

## Abstract

Disability might be relevant to decisions about life support in intensive care in several ways. It might affect the chance of treatment being successful, or a patient’s life expectancy with treatment. It may affect whether treatment is in a patient’s best interests. However, even if treatment would be of overall benefit it may be unaffordable and consequently unable to be provided. In this paper we will draw on the example of neonatal intensive care, and ask whether or when it is justified to ration life-saving treatment on the basis of disability. We argue that predicted disability is relevant both indirectly and directly to rationing decisions.

## Introduction

Every day, in intensive care units around the world, medical professionals withhold or withdraw life saving treatment (LST) from disabled critically ill newborn infants (Weiner et al. 2011; Verhagen et al. 2010; Singh et al. 2004; Wilkinson et al. 2006; Fontana et al. 2013). Concern for a child’s quality of life lies behind as many as 1/3 to ½ of decisions to limit LST in intensive care units in Australia, Northern Europe and the United States (Fontana et al. 2013; Verhagen et al. 2010; Wilkinson et al. 2006). Although these decisions remain controversial, they have been endorsed by professional guidelines and the courts (Wilkinson 2013a). The essential ethical basis for such decisions has traditionally been based on two premises: first that it is permitted to avoid or stop treatment that is not in a child’s best interests; second, that in some circumstances the prediction that a newborn infant will be disabled (if they survive) appears highly relevant to a child’s best interests (Wilkinson 2006). Future disability may affect the balance of benefits and burdens for the child (Wilkinson 2013a). Disability may mean that a child requires highly unpleasant and burdensome treatment to stay alive (for example if a child is unable to breathe by themselves). Alternatively, the disability itself may be sufficiently unpleasant that it causes substantial burdens (for example, a child with severe congenital blistering of the skin (epidermolysis bullosa) or distressing compulsive self-mutilation (Lesch Nyhan syndrome)). Finally, the disability may substantially reduce the benefits of life-saving treatment for the child. Indeed in some circumstances (where the child would have no conscious awareness), the disability may mean that life-saving treatment appears to offer no benefit to the child at all (Wilkinson 2006).

Decisions about LST for newborn infants are usually made in conjunction with a child’s parents (Nuffield Council on Bioethics 2006; Gillam and Sullivan 2011). Parents have a key epistemic role to play in determining whether or not treatment is in a child’s best interests (Wilkinson 2010a). Arguably, parents’ interests should also be taken into account in decisions, because of the degree of overlap between the interests of the child and those of parents, and because of the impact of caring for the child on the family (Wilkinson 2010a, b). Future disability, particularly if severe, may significantly affect the interests of parents and other family members, and consequently affect decisions about LST that way (Wilkinson 2013a).

However, there is another important potential ethical consideration about LST for newborn infants—that of distributive justice and the need to ration expensive and scarce medical resources. Although almost all discussion about LST in newborn infants focuses on the child’s best interests, (and perhaps on the interests and wishes of parents), resources are of central importance in intensive care, for newborn infants as for older patients (Camosy 2010; Wilkinson 2013b). Sometimes it is necessary to ration potentially beneficial LST on the grounds of distributive justice. But is disability relevant to such decisions? Is it justified to deny life-saving treatment to disabled newborn infants (on the grounds of resources), when the same treatment would be provided to non-disabled infants? Or would such a decision represent a form of ethically problematic discrimination?

### Assumptions

For this paper we will focus explicitly on the role of disability in rationing in newborn intensive care. We will set aside the interests of the child and those of parents. Sometimes rationing questions, and best interests questions converge. It may be ethical for clinicians to decline to provide some ostensibly futile treatments both on the grounds of the child’s interests, and on the grounds of distributive justice (Wilkinson and Savulescu 2011). Rationing questions and the interests of parents may also converge. The consequences of providing LST may be considerable for both parents and for society. For the sake of this paper, though, we are interested in the most difficult divergent cases—where clinicians are potentially declining to provide treatment that is both beneficial and is desired by parents (Box 1). We will assume, for the sake of argument, that treatment would probably be in a child’s best interests.

**Box 1:** The example of Spinal Muscular Atrophy type 1—is it discriminatory?

Spinal muscular atrophy type 1
A short while ago, one of us wrote an editorial on the ethics of providing long-term mechanical ventilation for newborn infants with severe type 1 spinal muscular atrophy (SMA) (Wilkinson and Gillam 2013). SMA is a rare genetic disorder that leads to degeneration of motor neurons. There are several different sub-types of SMA, but the most severe, (type 1), causes profound muscle weakness in infancy. Infants may appear relatively normal at birth but develop progressive weakness of all of their muscles leading to low muscle tone, poor feeding, and respiratory failure by about 6 weeks of age. Infants usually end up receiving mechanical ventilation because their breathing muscles are too weak for them to be able to breathe on their own. The ventilator solves that problem, however, the muscle weakness continues to progress, and such infants usually develop a version of the locked-in syndrome. They are paralysed, and often unable to move any muscles except their eyes. To survive, such infants would require long-term invasive breathing support. Should such treatment be provided? Until recently, this was not an option. Once genetic or other tests confirmed the diagnosis, the usual course in most parts of the world was to withdraw LST and to allow the child to die (Gray et al. 2013; Ryan et al. 2007; Hardart and Truog 2003). However, in recent decades there has been debate about whether or not these infants, or a sub-set of them, should be offered the option of tracheostomy and long-term mechanical ventilation. Some parents have refused to consent to withdrawal of treatment and consequently treatment has continued against medical advice (Jonas 2007; Ryan et al. 2007). In some parts of the world, for example in France, there is experience with providing long term respiratory support including home mechanical ventilation to infants and children with SMA type 1 (Ioos et al. 2004; Rul et al. 2012; Bach et al. 2003)
Debate about provision of long term mechanical ventilation for infants with SMA type 1 has focused on whether or not treatment is in a child’s best interests (Gray et al. 2013; Hardart and Truog 2003; Rul et al. 2012). There are different views, yet, it seems plausible that such treatment could be a net benefit for the child (Wilkinson and Gillam 2013). French experience indicates that surviving children are able to communicate, gain an education, and develop relationships (Rul et al. 2012). Adults with locked-in syndrome in a number of studies have reported a subjective quality of life that is within the normal range (Lulé et al. 2009; Snoeys et al. 2013), and usually a preference to continue living (Bruno et al. 2011) However, there has been little attention to the question of resource allocation in SMA. In the editorial, one of us wrote
“One estimate (now some years old) of the cost of providing invasive ventilation in this condition was in the range of $100,000–200,000 per year. If this estimate is correct, there is no way that the treatment can be justified. … In a recent study, health utility for ‘muscular dystrophy and spinal muscular atrophy’ was assigned a mean utility of 0.36 (interquartile range 0.11 to 0.65) based on care givers’ reports. If the lower of these estimates were used (given that ventilator-dependent SMA type 1 is at the most severe end of the muscular dystrophy/SMA spectrum), this would yield a cost per [quality adjusted life year] QALY of $900 000.” (Wilkinson and Gillam 2013)
After writing this editorial, the author received an email from a parent and advocate for children with disability. The correspondent asked
“Is it appropriate to use QALY for …a cohort of patients who are disabled when the treatment options are death or life? Could a QALY assessment result in the decision to offer a certain treatment/drug etc to a non-disabled cohort but withhold the same option for a disabled cohort? If QALY can be applied to a disabled group … resulting in non treatment and death, isn’t it extremely discriminatory?”^a^

^a^Personal correspondence, February 2014. This quotation is reproduced with permission of the correspondent

Next, we accept what seems almost incontrovertible, that health resources are limited, and therefore that decisions have to be made about which treatments to provide, and which patients to treat. Of course, some patients might be able to overcome resource limitations because they can afford to pay for the treatment themselves. Then, even if the treatment cannot be afforded out of public funds, it would still be possible for them to receive it. Alternatively, perhaps there are some countries that are so wealthy that they do not need to impose any limits on medical treatment. Those situations might raise different issues about fairness (is it fair for wealthy patients to be able to obtain treatment that is unavailable to poorer patients), but let us set those aside. We still need to know what to do in the far more common situation globally where resources are finite (Miljeteig and Norheim 2006; Miljeteig et al. 2009, 2010).

Some treatments are so expensive that it may not be possible to provide them to any children, even if they would be of benefit.^1^ In those cases, disability is not likely to be relevant. We will assume therefore, that treatment would be sufficiently beneficial, relative to its cost, to justify providing it to infants who are not predicted to be disabled.

We are going to focus on *life*-*saving* treatment, treatment that will make the difference between life and death for a newborn infant. In some cases non-provision of a medical treatment will not result in death, rather it may lead to an infant surviving in a more impaired or compromised state. We will not discuss those cases further. Nor will we discuss the questions that arise when there is a choice between life-saving treatment and non-life saving treatment.^2^

Finally, it is perhaps worth noting at this stage that the aim of this paper is to assess *whether* disability should play a role in decisions about rationing life-prolonging treatment. If the answer to this question is in the affirmative, there are further questions to be asked about *how* rationing should be undertaken, whether it should occur on the same basis for non-life prolonging treatment, and whether infants should be treated differently from older children and adults. Those questions we will have to set aside for another day.

If disability is relevant to rationing, it might be relevant in one of several ways. It could affect the costs of treatment or the benefit of treatment. If it affects the benefit, it could affect the probability or the duration or the magnitude of benefit. We will consider each of these in turn.

## The cost of treatment

### Should doctors consider the costs of potential medical treatments?

In neonatal intensive care, should it make a difference how much a potential treatment would cost?

One view might be that doctors should only consider what is best for their patient, they should not take into account the cost of medical treatment. Yet, it would seem wrong for doctors in a public health system to ignore the costs of treatment. Imagine that a neonatologist has a choice between two antibiotics for a patient that are equally effective, antibiotic A and antibiotic B. However, antibiotic B costs ten times as much as antibiotic A. In that situation it would be a misuse of finite, and precious health resources for the doctor to choose antibiotic B.

A more difficult problem faces doctors when they face the same type of choice but with different patients. If they had a treatment that would be just as effective for two different patients, but would be much more costly for infant B than for infant A, which should they choose? We might like to avoid the question by treating both infant A and infant B, but sometimes that will not be possible. If there are no other relevant differences, and it is only possible for the doctor to treat one patient, it seems ethical for the doctor to treat infant A rather than infant B. One important reason is that treating infant B might be expected to lead to other infants (perhaps C, D etc.) not receiving treatment at all. For example, the more expensive treatment might be a longer intensive care stay. If there are limited intensive care beds, treating infant B for ten times as long as infant A potentially means that other infants are unable to be admitted to intensive care and die as a result. To put it starkly, when there are fixed resources, cost equates to numbers of lives. The doctor’s dilemma is equivalent to John Taurek’s famous ‘Lifeboat’ example (Taurek 1977). Faced with a decision between saving one stranger or five strangers, most people would intuitively choose to rescue five (Otsuka 2006).^3^

However, some people may still feel that doctors should not be responsible for managing the health budget. They should only look after the best interests of patients in front of them. It may be acceptable for treatment to be rationed, but doctors should not be involved in rationing at the bedside. The obvious solution would be to ensure that rationing decisions are made by other people working in a hospital (triage officers, or administrators or finance officers), or at a policy level. For example, a hospital policy might state that antibiotic B should not be provided, or that patients with B-type problems should not be admitted to intensive care. When we are thinking about triage officers or policy makers it does not seem to make a difference whether we are talking about the same patient, or different patients. If the health budget is finite, treatments that are less expensive (but have the same benefit) should be preferred over treatments that are more expensive.

### Discrimination against expensive health conditions

One potential concern about cost as a factor in rationing health treatments is that this would discriminate against patients with expensive health problems (Harris 2010). It is no fault of the newborn infant B that they happen to have a condition that is expensive to treat. Some might feel that it would be unfair to have a policy that excludes infants like B from life saving treatment.

The first claim, that this would be discrimination, appears in one sense irrefutable. It is in the simple sense that to discriminate is to choose. It is discrimination to provide life-sustaining treatment to some patients but not to others. But in the example as described, doctors have no choice but to choose—they must discriminate in this sense. The question is whether this discrimination is unjust. Is the choice based on ethically relevant considerations (in which case it is arguably just) or on irrelevant considerations (in which case it is not)? (Arneson 2006). It would be wrongfully discriminatory to choose not to treat infant B on the basis of race, or parents’ religion/political views. However, the cost of treatment *does* appear to be a relevant consideration, not least because of its implication for the number of lives saved. Discrimination on the basis of cost is not unjust. Indeed justice would seem to require that we save five rather than one (and thus choose the less expensive treatment).

What of fairness? We accept that it is unfair for an infant to die prematurely for want of an available life-saving medical treatment. But it would appear to be equally unfair for infant A or infant B to die, (or C or D etc.). It would not become more fair if we selected A or B by chance, by tossing a coin. Indeed, given that this would give us a 50 % chance of choosing the more expensive treatment (and thereby potentially leading to more unfair premature deaths), such a strategy would appear more unfair than a decision to choose the less expensive treatment.

To conclude then, both justice and fairness permit, even require, the use of cost in prioritizing the use of limited life saving resources.

### Disability and the cost of medical treatment

Could disability affect the cost of medical treatment? Some disabilities in the newborn period can only be treated with highly expensive life-saving treatment. For example, a newborn infant born with severe renal failure may be able to be saved only with the provision of long-term renal dialysis. A newborn infant with severe infantile cardiomyopathy may be able to be saved only with a heart transplant. We have seen that doctors^4^ are justified in making rationing decisions on the basis of the cost of treatment. It appears, then, that they would be justified in excluding some disabled infants from life-saving treatment because treatment is too costly.

The examples above represent acutely expensive life-saving treatment. However, some newborn infants may have health conditions that are highly expensive in the long-term rather than in the short-term. Should long-term health costs be considered? One reason to consider them is that it does not make any sense to only include the short-term consequences of decisions. We saw above that doctors should choose the less expensive treatment A over treatment B. However, imagine now that treatment A needs to be given life-long, such that it ends up being 10× more expensive than treatment B. It would be extremely short-sighted to choose A merely because it is cheaper now, while ignoring the long-term costs.

Some disabilities may have considerable long-term costs. In the example of SMA type 1, the long-term care costs may be many times greater than the acute care costs—depending on how long a child lives. Short-term respiratory support for an infant with SMA is not particularly expensive, no more so than the cost of ventilating an infant with bronchiolitis or a premature infant. The costs accrue, however, the longer that treatment continues. For infants with renal dialysis, there are the costs of ongoing dialysis, as well as the potential cost of transplantation. These disabling conditions are expensive because treatment of their underlying condition (renal or respiratory failure) is extremely expensive over a long period, perhaps as long as they live. Is it reasonable for neonatologists to take this into account? It appears to be, on the basis of arguments already outlined. At a global level, treatment for long-term renal failure or respiratory failure in infancy appears to lie outside what is affordable in many health care systems. Even if other life-saving treatments are available, infants with SMA or renal failure are often excluded.

## The benefit of treatment—part 1—quantity of life

### The probability of benefit

If doctors (or administrators or policy makers) are permitted to take into account the costs of treatment, should they also factor in the benefit of treatment?

It is obviously justified for neonatologists to take into account the probability of benefits of treatment when thinking about a single patient. Imagine that they have two life saving treatments for an infant, treatment C and treatment D. Imagine that treatment C had a 90 % chance of saving a patient’s life, while treatment D had only a 9 % chance of saving their life. It would be unethical for the doctor to give treatment D.

The situation might be thought to be similar if the doctor were treating two different *patients*, patient C and patient D. Patient C has a 90 % chance of survival with treatment, while patient D has only a 9 % chance of survival. For a concrete example, patient C might be a premature infant born at 28 weeks gestation, while patient D might be an infant born at 22 weeks gestation (Wilkinson 2012). If the doctor is only able to treat one of these patients (perhaps because there is only one intensive care bed available) it appears justified to treat infant C (the 28 week gestation infant) rather than infant D (22 weeks).

Some people might think that to be fair we should give every patient the same chance of receiving a life-saving treatment. It would be unfair to give infant D no chance of receiving treatment. Both C and D would benefit from treatment. We might think that they have an equally strong claim to be treated or an equally strong interest in being treated. The strength of an interest in treatment may depend upon our theory of interests (DeGrazia 1995). For example, a desire-based account of interests would see no distinction on the basis of probability. Although it is less coherent in the case of infants, for older individuals it is plausible that a patient’s desire for life-saving treatment would be independent of the probability of the treatment working. A patient with a 10 % chance would have just as strong a desire to be treated as a patient with a 90 % chance (Harris 1996). However, on this view it seems that we should give patient D treatment even if they have only a 1 % chance of surviving with treatment, while patient C has a 100 % chance of surviving. This conclusion is hard to accept.

We could avoid this conclusion by referring to an alternative account of interests. An objective account of interests might hold that an individual has a stronger interest in something that would yield a greater objective benefit to them. Plausibly, a reduction in the probability of objective benefit would lead to a reduction in the strength of interest. We should treat patient C.

Finally, if doctors decide to treat patients with a higher chance of surviving, this does not need to be based on the view that they have a stronger claim to treatment. Rather it is based on the idea that limited health resources should be distributed to satisfy as many equal claims or interests as possible. Just as with cost, probability equates to numbers of lives saved. Treating 10 patients with condition C will save nine lives, while treating ten patients with condition D will save only 1 life. We can save nine or one. Providing LST to patients with higher chances of survival makes it more likely that patients’ desires to live will be fulfilled.

### The small differences argument

When the differences in chance of benefit are large, the argument in favour of treating the patient with a higher chance appears strong. Yet, when the differences are small, it is less clear what doctors should do. For example, if one patient (E) has a 22 % chance of survival, while another (F) has a 20 % chance of survival, perhaps it would be wrong to just treat the patient with a higher chance. One concern might be that doctors are not very good at determining probability of survival for patients. Doctors might be mistaken in believing that E has a better chance. The patients could actually have the same chances. Or patient F may, in actual fact, have a greater chance of benefit. It is important that doctors are careful to use the best available evidence. If the evidence is reasonable, they will reach the correct conclusion more often than they will reach an incorrect conclusion. It might be, though, that often the evidence for working out the chance of survival is weak. In that case, doctors should be careful not to be too strict in applying rationing on the basis of estimated benefit. Because E and F have *similar* chances of benefit, they should actually receive equal chances of receiving life-saving treatment. In a different context Aristotle noted that “it is a mark of an educated person to look in each area for only that degree of accuracy that the nature of the subject permits.”(Aristotle 2000: 4–5). Following Aristotle, we should be conscious of the uncertain nature of prognostication and avoid overly precise distinctions (McMillan et al. 2013) (Savulescu 2002).

On the other hand, sometimes our estimates of benefit will be more certain. Or they may be much further apart. If doctors estimate that F has only a 1 % chance of survival, while E has a 100 % chance of survival, we should treat E rather than F because it is highly unlikely that F’s chance of survival is actually equal to (or better than) E’s chances.

### The duration of benefit

Similar arguments would appear to favour choosing treatments (or patients) with a longer duration of survival. In an individual patient it would clearly be preferable to choose a treatment that led to a patient surviving for a longer rather than a shorter period.^5^ We could imagine that treatment H would lead to an infant dying in childhood (at age 7 say), while treatment G would lead to the same infant surviving to age 70. The doctor should choose treatment G. If there were a choice between different patients, G and H, with different life expectancies after life-saving treatment, it appears to be appropriate to choose to treat the former patient on the same grounds.

There might be similar objections to deciding between patients on the basis of duration of benefit, and similar responses. So, it might be claimed that patient H has just as strong a claim or interest in treatment as patient G. But this view again risks absurd conclusions. If doctors had to choose between saving the life of an infant who would live to a full adult life expectancy compared with one who would live for only one week or one day, should they just toss a coin?

When the predicted duration of survival is very low, there may be a question about whether or not life-saving treatment would actually constitute a net benefit. In this way duration of benefit and probability of benefit can overlap. For example, if a newborn infant were required to undergo complicated and painful surgery for a complex malformation but were only able to survive for a few months, some might question whether it was worth it, whether the benefit outweighed the burden. It is difficult to know how long infants would need to survive in order to outweigh the burden of a particular negative intervention. However, it appears plausible that the probability of benefit from LST would fall, the shorter the period of survival.^6^ Drawing on the above argument about precision in prognosis and rationing, we should also note that small differences in predicted survival should not necessarily lead to exclusion from LST.

### Disability and probability or duration of benefit

Rationing LST on the basis of probability or duration of benefit appears highly likely to affect some disabled infants.

For example, some disabling conditions are associated with death in childhood or early infancy. Children with Tay Sachs disease survive on average until the age of 3 years (Bley et al. 2011). Children with severe cerebral palsy and mixed motor, cognitive and visual disabilities often die by the age of 13 years (Hutton et al. 2000). Other disabling conditions are associated with a reduced chance of survival. For example, in population studies, only 10 % of infants with trisomy 18 survive past the first year of life (Wang et al. 2011). Severely disabled children admitted to paediatric intensive care had a 70 % survival rate, compared to 90 % in children with mild or no disability (Namachivayam et al. 2010).

We have found it is just to discriminate on the basis of cost, duration and probability of outcome from life saving treatment. When disability affects one of these three variables, it is just to include it to the to the extent that affects that variable.

## The benefit of treatment—part 2—quality of life

### The magnitude of benefit

What if there were no difference in the costs of treatment, nor in the probability of survival, nor in the duration of benefit? Should the child’s future quality of life be considered? Would the fact that the infant will be disabled, be *directly* relevant to rationing decisions?^7^

The example of SMA type 1 is highly relevant here. We might compare it with another condition that leads to long-term dependence on mechanical ventilation (Box 2).

**Box 2:** The example of Congenital Central Hypoventilation Syndrome. Should it receive priority for treatment over SMA type 1?

Congenital Central Hypoventilation Syndrome (CCHS) is a rare genetic condition in which there is abnormal regulation of breathing during sleep (Trang et al. 2005). Affected infants develop abnormal slowing and pauses in their breathing during sleep. If untreated, the severe form of this condition leads to death within the first month or two of life. However, with treatment, children may survive for years (Trang et al. 2005). During the day, while awake, these children have completely normal function. They do not require any assistance with their breathing. They are able to run, walk, communicate, attend school. Overnight, however, they must be attached via a tracheostomy to a ventilator to maintain regular breathing and to stay alive. Should CCHS be treated differently from SMA type 1? It is plausible that the costs of treatment and the probability and duration of survival could be similar.^a^ However, on many accounts of quality of life, the quality of life of a child with CCHS is much higher than a child with SMA

^a^Older children with CCHS may be able to be managed with non-invasive ventilation, and thus no longer require tracheostomy (Ramanantsoa and Gallego 2013). It is possible therefore that there are in reality differences in prognosis between CCHS and SMA type 1. For the sake of argument though, let us assume that these factors are identical—or at least similar enough that on grounds of cost/duration/probability the two conditions should be treated similarly

Again, if we think about an individual patient, doctors make decisions all the time, choosing between treatments on the basis of quality of life. For example, imagine that a doctor has to choose between two life-saving treatments I and J for a single infant, Betty, perhaps different surgical techniques. The treatments cost the same amount. They have the same chance of saving Betty, and she will live for the same amount of time. However, if the doctor uses surgical technique J, Betty will live to adulthood but be affected by cerebral palsy and cognitive impairment. If the doctor uses technique I, she will live to adulthood with no impairment. In this choice, it is clear that the doctor should provide treatment I. It seems uncontroversial that at least this sort of quality of life assessment is justified.

What about a situation where there are different patients, Ingmar and Julian, requiring treatment? Prima facie, it appears that the same considerations would apply. Drawing on the same line of argument as used above for costs, probability and duration of benefit, it appears that quality of life could be a reason to choose to treat Ingmar rather than Julian, in a situation where only one patient could be treated. It could be justified, therefore, to selectively provide treatment to a non-disabled over a disabled infant (Savulescu 2001).

Again, this would appear to hold for large differences in quality. Where there are small differences, as before, it seems unjust to make too discriminating judgements. Imagine Ingmar has a sunny temperament and Julian is flat, more melancholic. It would be wrong to give priority to Ingmar, even if we believed it was better to be happy than sad. Whether disabilities like blindness and deafness should be treated as melancholia or severe physical and cognitive impairment is a difficult question to which we will return. For the present, it seems reasonable to take into account significant impairments in quality of life prognosis in allocation of life saving treatment.

### The one-to-many analogy

The above represents an argument from analogy. More formally we could set it out as follows:

1. Two life saving treatments T1 and T2 differ in characteristic X to a degree Y.
2. Two patients P1 and P2 if provided with life-saving treatment differ in characteristic X to degree Y.
3. If a doctor were treating a single patient, differences in characteristic X to degree Y would provide a moral reason to provide treatment T1 rather than T2.
4. If characteristic X (to degree Y) is relevant for individual patients, it is also relevant for decisions about multiple patients.
5. Difference in characteristic X (to degree Y) provides a moral reason to provide treatment to P1 rather than P2.

Some may reject the one-to-many analogy. For example, John Harris apparently rejects the fourth premise on the grounds of patient preferences (Harris 1987, 1996). A single patient may have a strong preference for survival without impairment, and consequently for T1 over T2. Yet our two different patients P1 and P2 may have, indeed are likely to have, equally strong preferences for life over death. If we are providing life-saving treatment in order to respect patient interests, and a patient’s interest in LST is based on their preference for that treatment, it appears that the one-to-many analogy would fail (Harris 1987; Sinclair 2012).

However, as noted above, Harris’ argument would also reject the one-to-many analogy where characteristic X is the cost of treatment, or the probability of survival, or the duration of survival. On Harris’ account, a patient with very low (<1 % say) chance of survival, predicted to survive for only a period of days, and only at vast expense should have an equal chance to receive a LST as a patient who has a 100 % chance of survival for many years if provided with an inexpensive LST. Moreover, when we are talking about newborn infants, our basis for choosing T1 over T2 cannot be based on their preferences. They do not have a preference for either treatment.^8^ If premise three holds in the case of newborn infants, it does so because characteristic X represents a morally salient reason to prefer state T1 over T2. We might refer to rational preferences, and to what an infant would have reason to prefer. Or we could refer to objective constituents of wellbeing. Although there remains considerable philosophical disagreement about theories of wellbeing, it appears likely that any reasonable individual would choose treatment that offered longer survival over shorter survival,^9^ a higher chance of survival over a lower chance of survival, or unimpaired survival over significantly impaired survival. High level of agreement with premise three provides us with evidence about patient preferences. However, it also supports the view that in general life is of benefit, and that in general impairment reduces wellbeing. Accordingly, the one-to-many analogy gives doctors or policy makers or triage officers a guide as to the types of benefit that they should consider when using limited medical resources. Medical treatment should be distributed to secure across a population as many as possible of the benefits that individuals would choose for themselves.

### Quality of life and a life worth living

Some people would object to a policy that treated patient I rather than patient J because patients who have cerebral palsy have lives that are worth living. They might be concerned that a decision not to treat patient J is because of a mistaken belief that life in a wheelchair or life with cerebral palsy is worse than nothing. However, as we saw when doctors are making decisions for an individual patient, the reason to choose treatment I over treatment J does not involve an assessment of whether life in a wheelchair is worth living. It is based on an assessment of whether life with full ability to walk is in general better for the individual than not being able to walk. Indeed, for the purposes of this paper we are confining ourselves to situations where treatment would probably be in the child’s best interests. The question is whether disability would justify rationing beneficial and desired treatment.

Alternatively, critics may argue that states of disability are not *as bad* as they are commonly believed to be by individuals who are not disabled. They might point to the large body of sociological literature supporting the ‘disability paradox’ (Albrecht and Devlieger 1999). When individuals with significant disabilities are surveyed about their level of happiness, their responses are often far more positive than others expect. For example, children with cerebral palsy report general levels of satisfaction that are similar to children without cerebral palsy (Dickinson et al. 2007). Even patients with the most severe form of physical disability, locked-in-syndrome, report generally being happy (Bruno et al. 2011). We agree that views about disability are often unduly negative, and that states of disability may not be as bad as they appear to the non-disabled. However, none of this would change the fundamental reason for rationing and the one-to-many argument. Even if life with cerebral palsy is not as bad as feared, a doctor would have strong reasons to choose treatment I over treatment J.^10^

Some people might argue that the reason that life with cerebral palsy is not as good as life with a full ability to walk is because of the way that our society is arranged (Amundson and Tresky 2007). For example, if people were fully accepting of people with disability, if there were more lifts, better designed cars, and public transport, adequate support in the community etc. that there would be no disadvantage in being in a wheelchair. It is clear that all of those things would be helpful for people with disabilities. It would be good to provide them. However, those arguments do not mean that in the individual patient case we should choose treatment J over treatment I. Even if our society were fully committed to reducing disadvantage to disabled people, it may take some time for those interventions to take effect. Moreover, even if all of those steps to reduce disadvantage had been taken there would still likely be reasons for the patient to prefer to survive not in a wheelchair, to prefer treatment I, in the way the world is likely to be. In addition, there are some disabilities that would lead to some disadvantage even in a perfectly just, accommodating, supportive society. For example, consider a child with SMA type 1; no amount of change to society is going to make survival with SMA type 1 as beneficial for the individual as survival with completely normal function.

### Quality of life and moral worth

One concern about the use of quality of life (and perhaps duration of benefit) in rationing is that such decisions represent a devaluation of those with disability. Even if there is no question whether such lives are worth living, the implication of our argument could seem to be that certain states of disability are less *worth saving*. Dan Brock writes:

We should avoid giving weight in [prioritizing life-sustaining treatment] to the effects of disabilities on the patient’s quality or length of life because that would imply that these effects and the disabilities that cause them reduce the value of the treatments and the lives they sustain… [t]his would conflict with the equal moral worth of persons…(Brock 2005: 98).

There are several responses to this concern. First, it appears clear in the case of treatments I versus J for Betty, that reduced quality of life *would* reduce the value of treatment J for Betty. But that does not imply that Betty’s *life* would be less valuable if she had received this treatment. The aim of healthcare and distributive justice as we have described it, is not to maximise social value; it does not represent an attempt to save the lives of those with the greatest potential to contribute to society, financially or otherwise. Rather, the aim of providing healthcare is in order to increase patients’ wellbeing. If we are forced to provide life-saving treatment only to some of the patients who we could save, we should choose more valuable over less valuable treatments, but not more valuable over less valuable lives. When we provide LST preferentially to patients who are less likely to survive, or whose treatment would be less expensive, that does not mean that those who would survive for a shorter period, or who would require expensive LST have any less moral worth. We would save both groups of patients if we could.

The relationship between disability and chance/duration of benefit or cost of treatment is contingent. In some circumstances disabled patients may receive lower priority for treatment on the basis of these factors, while in other circumstances they will not. However, if quality of life is incorporated into rationing, disability may be directly relevant to resource allocation. Then, even if we are correct to emphasise that rationing decisions would be based on the value of *treatment*, not the value of lives, those who are disabled may feel devalued because they are likely to receive a lower priority for treatment.

There are two related questions. The first question is this: when we are allocating scarce resources, how much weight should be given to equality, compared with the benefit of treatment? One reason why the answer about treatment for an individual patient may be different from the answer that we give when deciding between multiple patients, is that there is a competing moral consideration.^11^ If we give substantial weight to equal treatment of persons, we should toss a coin to decide between treating a newborn predicted to survive a week or month, or one who could survive for a normal lifespan. More plausible, perhaps, is a view we should give some weight to equality, but that this must be balanced against benefit. We should usually treat patients equally where there are only modest differences between them in their predicted duration of survival or future quality of life.^12^ However, where there are large differences in benefit this will outweigh equality. If we had to choose between providing long term ventilation for a newborn with CCHS, compared with one with severe SMA, we should choose the infant with CCHS.

The second question is how much weight we should give to the social context in which allocation occurs and the way in which society understands allocation. We have argued that giving preference to treatments that are likely to secure a greater benefit does not necessarily devalue those individuals who are denied treatment. Yet, if there is a widespread misperception in society that disabled individuals have lives that are not worth living, or are less valuable,^13^ and if resource allocation would reinforce that perception, we should potentially take this into account. We will return to this possibility shortly.

### Probability of harm and quality of life

For this section we have stipulated that the probability of survival is the same between different treatments that we could provide. Yet, probability might be relevant to quality of life in a different way. We noted earlier that we are referring to conditions where LST would probably be in a child’s best interests. But when we are talking about individuals with very severe disabilities, such as SMA type 1, there are likely to be different views about whether or not the benefits of life-sustaining treatment outweigh the burdens (see Box 1). Some people may believe that in fact it would be a harm to these individuals to use intensive medical treatment to prolong their lives (Gray et al. 2013; Isaacs and Kilham 2013). It is very difficult to be sure. For conditions where the individual is unable to communicate we can never know exactly what life is like from their point of view. Although our view is that treatment for a child with SMA type 1 or other similarly severe conditions would probably be in their interests (Wilkinson and Gillam 2013; Savulescu 2013), it does seem possible that life-saving treatment will overall be a burden to them. That could be, for example, because of extra medical problems that arise. It might be because of the burden of extra unpleasant and invasive medical treatments. It might be because in the absence of an ability to communicate, caregivers underestimate how much the child is suffering. If that is right, that there is a chance that patients who are severely disabled will not benefit (and will be harmed) by providing life saving medical treatment, then we are back to talking about the probability of benefit. We already decided that it would be reasonable to prefer a 90 % chance of benefit (saving life) for C over a 9 % chance for D. On the same basis, it would be reasonable to treat a patient with CCHS rather than a child with SMA type 1 because the former has a higher chance of net benefit.

There are two extra things to say about this ‘chance of benefit/harm’ argument. First, it is unlikely that doctors or policy makers will be able to confidently put a number on the probability of benefit. But we do not need to do that to be able to compare the chances for infants with SMA and CCHS. We do not know the exact numbers, but we do know that the chances are very different for them—sufficiently different that it would be justified to treat a child with CCHS rather than one with SMA if forced to choose. Second, this chance of benefit argument applies only to cases of very severe disability. If we were considering LST for an infant with Trisomy 21 (Down syndrome), there would be very little chance that his life, after intensive care, would be not worth living. Similarly, for blindness, deafness, diplegia, mild or moderate physical or intellectual disability; these disabilities, though significant, are not so severe that it is at all likely that the burdens would outweigh the benefits in their lives. Correspondingly, the chance of harm argument would not apply.

## Disability, discrimination and death

We have argued that disability is relevant to resource allocation and rationing decisions for newborn infants. It is relevant indirectly,^14^ through its relationship with other factors that are commonly, and (mostly) uncontroversially, thought relevant to resource allocation—the cost of treatment, the probability of survival, and the duration of survival. Quality of life is relevant directly because of the relationship between wellbeing and disabling states, and indirectly (for infants who are severely disabled) because of the probability of overall harm.

Accordingly, it would be justified in our view to ration life saving treatment on the basis of disability. Even those who reject direct discrimination on the grounds of quality of life and its relationship with wellbeing should still accept the indirect relevance of disability to cost, duration or probability of benefit.

How should rationing take place? Although this is not the main focus of this paper, here is a preliminary answer. First, it is critical that decisions about treatment allocation should be based on objective data, and avoid bias. Doctors sometimes have views about the chance of survival and degree of disability that are not borne out by up to date evidence (Wilkinson et al. 2014). They have been reported to rate quality of life of disabled patients more negatively than patients themselves (Saigal et al. 1999; Rothwell et al. 1997). This does not mean that rationing should be avoided—but it should be done with care.

Next, the small differences argument suggests that rationing should not be based on fine-grained distinctions between different disease states or different prognoses. Prioritisation would be most likely to be justified where the difference in predicted outcome is large. Plausibly, distributive justice is about giving as many people as possible a decent chance of a decent life (Savulescu 2009). A ‘sufficientarian’ approach to rationing means that provided someone’s quality of life is above a relevant threshold, or would be with treatment, they would be an equal candidate for lifesaving treatment. For example deafness and blindness may be disadvantages, but if they represent points above the threshold—patients who are deaf or blind would stand an equal chance of lifesaving treatment with those who are sighted or hearing.

Where we draw the threshold for providing LST will depend on the level of resources that are available as well as the cost of particular treatment. It is beyond the scope of this paper to determine where this threshold for a ‘decent life’ lies. Nevertheless, the arguments summarized above suggest that certain severe disabilities may fall below the threshold for expensive treatments. That might mean, for example, that long term mechanical ventilation is not made available to children with SMA type 1, but is made available to children with CCHS. It might mean that some children with severe disabilities are not offered the option of cardiac transplantation (Savulescu 2001). Rationing is most likely to affect moderate to severe cognitive disabilities and severe to profound physical disabilities, since these plausibly have the greatest impact on the wellbeing, the highest chance of overall harm, and the highest associated cost of treatment (Wilkinson 2013a).

We might choose not to ration in the way described above. Although it would be ethically acceptable and just to make decisions at a policy level that directed life saving treatment to patients without severe disabilities, it would be open to society to decide otherwise. It is always open to us to do more than we are morally required to, to provide more than justice requires. There are several potential reasons for such a policy. One reason would see the provision of intensive life saving treatments to severely disabled individuals as a form of compensation for the ways in which they have suffered disadvantage in the past as a result of their medical problems, and as a result of society’s attitude towards the disabled. Alternatively we might ask policy makers not to take disability into account (even though they would be justified in doing so) in order to promote the importance of care for the most disadvantaged in society and to counter pervasive devaluation of disabled lives. It would be a form of affirmative action. A very pragmatic reason to avoid rationing on the basis of disability might be due to a concern about the impact of such rationing on other decisions about LST. For example, some parents might resist decisions to withdraw LST on the basis of a child’s best interests because they believe (mistakenly) that doctors are covertly rationing treatment.^15^

If society were to take affirmative action in its allocation of intensive life-saving treatment, we should be aware of the consequences. Unless health budgets are increased considerably to reflect this policy it is inevitable that provision of expensive treatment to such patients will lead to denial of life-saving treatment to other patients with a higher chance or magnitude of benefit. It will lead to patients dying who could have been saved. In well-resourced health care systems there may be sufficient neonatal intensive care beds, and doctors may be able to avoid (most of the time) difficult questions about which life to save. However, this is a luxury that health professionals in many neonatal intensive care units around the world do not have. They cannot provide mechanical ventilation or dialysis or cardiac surgery or transplantation to every infant who might benefit. What should they do then?

In such situations rationing is not only just, it is just inevitable.

## Footnotes

1 Long-term mechanical ventilation, in the case example (Box 1), might be an example of such a treatment, since it appears to significantly exceed the standard cost/Quality Adjusted Life Year threshold, even in unimpaired children. However, long-term ventilation is regularly provided to children in a number of countries. The question is whether it is justified to decide which children it should be provided to, on the grounds of disability.

2 We accept that these questions are important, however, it is important to determine first whether disability is relevant to questions about life saving treatment. If it is not relevant in those cases, then *a fortiori* it will not be relevant to cases where there is a choice between life-saving and other treatment.

3 Taurek famously disagrees with this common intuition. However, a discussion of his views and the many responses to them (eg. (Parfit 1978)) is beyond the scope of this paper.

4 Or triage officers, or policy makers etc.

5 Assuming, of course, that the patient experienced more positives than negatives during the period of their longer life.

6 Beyond a certain point the opposite may not be true. The probability that life saving treatment would be beneficial may not increase any further once an infant is predicted to survive for at least a few years. For example, there may be no increase in the probability of benefit if the infant were to survive for 25 or 30 or 35 years.

7 Some discussions of discrimination distinguish between direct and indirect forms. Indirect discrimination refers to decisions that are not intended to disadvantage a particular group, but nevertheless do so because that group is disproportionately affected by that decision. Direct discrimination is said to involve intentional treatment of a particular group in a different way (Altman 2011). Drawing on this distinction, rationing on the basis of cost/probability/duration of survival might be thought to indirectly discriminate, while rationing on the grounds of quality of life directly discriminates. Indirect discrimination might be easier to justify, yet it does not always mark a moral difference. Both indirect and direct discrimination could be justified in some circumstances—if they are based on ethically relevant considerations, or unjustified where they are not.

8 Some might hold on this basis that they do not have an interest in receiving LST, and consequently that it would not be wrong to decide to withhold both T1 and T2 from them. However, even those who hold this view would presumably feel that it would be impermissible to choose T2 over T1. This is plausibly because of the future preference/interest that they would have in having a longer life rather than a shorter life etc.

9 Assuming that they experience more positives than negatives in their longer life.

10 Indeed, imagine that a doctor were to choose treatment J over treatment I, and led to a child surviving with preventable cerebral palsy. The doctor might cite, in their defense, literature on the disability paradox. Yet it seems highly unlikely that this would lead to them being found not guilty of negligence. Indeed, it seems plausible that such evidence would be completely disregarded…

11 The one to many argument therefore provides a pro tanto moral reason, but not an all things-considered moral reason for interpersonal choices. (We are grateful to David Wasserman for highlighting this point).

12 In the ‘small differences argument’ as set out earlier, this was based partly on uncertainty. However, it is also justified because of the value of equal treatment.

13 For example, see (Downie 2012, National People with Disabilities and Carer Council 2009).

14 See note 7.

15 There is no empirical evidence (to our knowledge) to support such a fear. Anecdotally, however, some families do express such concerns, and cite them as a reason for requesting treatment, even when medical professionals counsel ardently in favour of treatment withdrawal.
